# The Overlooked Photochemistry of Iodine in Aqueous Suspensions of Fullerene Derivatives

**DOI:** 10.1021/acsnano.2c02281

**Published:** 2022-05-09

**Authors:** Madhusudan Kamat, Kyle Moor, Gabrielle Langlois, Moshan Chen, Kimberly M. Parker, Kristopher McNeill, Samuel D. Snow

**Affiliations:** † Department of Civil and Environmental Engineering, Louisiana State University, 3255 Patrick Taylor Hall, Baton Rouge, Louisiana 70803, United States; ‡ Utah Water Research Laboratory, Department of Civil and Environmental Engineering, Utah State University, 4110 Old Main Hill, Logan Utah 84322-4110, United States; § Department of Environmental Systems Science, ETH Zurich, Universitaetstrasse 16, 8092 Zurich, Switzerland; ∥ Department of Energy, Environmental, & Chemical Engineering, Washington University in St. Louis, 1 Brookings Drive, St. Louis, Missouri 63130-4899, United States

**Keywords:** Iodine, photochemistry, singlet oxygen, MS2 bacteriophage, cationic fullerene, C_60_, photosensitizer

## Abstract

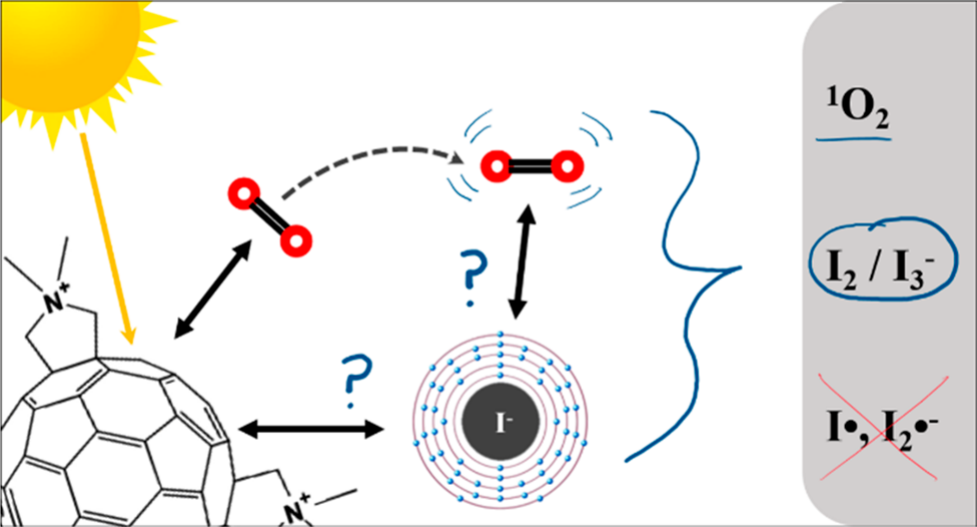

Fullerene’s low water solubility was a serious challenge to researchers aiming to harness their excellent photochemical properties for aqueous applications. Cationic functionalization of the fullerene cage provided the most effective approach to increase water solubility, but common synthesis practices inadvertently complicated the photochemistry of these systems by introducing iodide as a counterion. This problem was overlooked until recent work noted a potentiation effect which occurred when photosensitizers were used to inactivate microorganisms with added potassium iodide. In this work, several photochemical pathways were explored to determine the extent and underlying mechanisms of iodide’s interference in the photosensitization of singlet oxygen by cationic fulleropyrrolidinium ions and rose bengal. Triplet excited state sensitizer lifetimes were measured via laser flash photolysis to probe the role of I^–^ in triplet sensitizer quenching. Singlet oxygen production rates were compared across sensitizers in the presence or absence of I^–^, SO_4_^2–^, and other anions. 3,5-Dimethyl-1*H*-pyrazole was employed as a chemical probe for iodine radical species, such as I·, but none were observed in the photochemical systems. Molecular iodine and triiodide, however, were found in significant quantities when photosensitizers were irradiated in the presence of I^–^ and O_2_. The formation of I_2_ in these photochemical systems calls into question the interpretations of prior studies that used I^–^ as a counterion for photosensitizer materials. As an example, MS2 bacteriophages were inactivated here by cationic fullerenes with and without I^–^ present, showing that I^–^ moderately accelerated the MS2 deactivation, likely by producing I_2_. Production of I_2_ did not appear to be directly correlated with estimates of ^1^O_2_ concentration, suggesting that the relevant photochemical pathways are more complex than direct reactions between ^1^O_2_ and I^–^ in the bulk solution. On the basis of the results here, iodine photochemistry may be underappreciated and misunderstood in other environmental systems.

## 1 Introduction

Over the past three decades, fullerene materials, most commonly C_60_ or C_70_, attracted significant attention for their outstanding photophysical properties, among other characteristics. The finding that moderate functionalization of the carbon cage does not nullify the molecule’s photochemical properties opened a wide variety of prospective chemical, materials, and biological applications.^1,2^ Derivatization of fullerenes is particularly important in aqueous media, where the carbon allotrope would otherwise be practically insoluble. Even when researchers induced the formation of colloidal C_60_ aggregates, often termed nC_60_, the particles retained very little of the molecule’s innate photoactivity.^3,4^ Adding cationic functional groups to fullerenes proved to be a promising solution due to improved water solubility and photoactivity of aqueous aggregates.^5−7^ Synthetic chemists producing such molecules often opted for the use of iodide as a counterion, inadvertently complicating nearly all subsequent photochemical studies using cationic fullerenes.^8−11^ Until recently, important I^–^ photochemistry was overlooked by studies relating to health or the environment; three mechanisms warrant particular attention.

First, the so-called heavy atom (HA) effect is a well-documented phenomenon where atoms of elements with high atomic numbers can facility spin-forbidden electron transitions.^12,13^ Thus, heavy ions such as I^–^ can potentiate the production of singlet oxygen, ^1^O_2_, in many systems,^14^ such as in the case of some ^1^O_2_ sensitizing dyes (e.g., rose bengal and erythrosine). The mechanism of influence of HAs is generally attributed to the fact that large electron orbitals of the heavy atom make forbidden spin transitions possible, even at a distance of several nanometers,^15^ within photoexcited molecules.^13^ In this way, the HA can promote ^1^O_2_ production by allowing more efficient transitions from singlet excited to triplet excited states in sensitizers. Fullerenes, however, have innately efficient intersystem crossing (ISC) due to the curvature of their π–π bonding orbitals,^16^ so it is unclear to what extent the HA effect would increase the efficiency of ^1^O_2_ production from C_60_ derivatives. Alternately, the HA effect may reduce the net ^1^O_2_ yield by accelerating nonradiative decay of a triplet states directly^17^ or of anion-sensitizer exciplexes, as demonstrated recently with a porphyrin-containing sensitizer.^18^

Second, iodide may be oxidized by ^3^C_60_*. With a redox potential of 1.4 V_NHE_, I^–^ is thermodynamically susceptible to oxidation by triplet excited sensitizers. Since the redox potential of ^3^C_60_* is 1.5 eV, and most cationic-functionalized derivatives are only marginally lower,^1^ it is possible that, in aqueous solutions, I^–^ could be oxidized by excited state fullerenes to form C_60_^•–^ and I•. Studies employing cationic fullerenes have largely focused on producing ^1^O_2_ for water purification or photodynamic therapy.^3,7,8,10,11^ In these contexts, the formation of I• would have significant and largely overlooked implications. I• present in aqueous solution can lead to the formation of I_2_^•–^ and I_2_,^19^ which exists in equilibrium with I_3_^–^ and HOI.^20^ Further, C_60_^•–^ reacts with O_2_ to form superoxide radical anion (O_2_^•–^),^3^ providing another pathway to I_2_ formation: H_2_O_2_ is generated via superoxide dismutation reactions,^21^ then the reaction of peroxide with I^–^ forms HOI. The relevance of the presence of these radicals is immediately apparent for the biological and environmental systems studied for cationic fullerenes; estimations of the role of photosensitized ^1^O_2_ during toxicity assays, disinfection processes, or photodynamic therapy applications are likely inaccurate.

A third overlooked photochemical pathway is the direct reaction of ^1^O_2_ with I^–^. The ^1^O_2_ formed during C_60_ photosensitization can react directly with I^–^ to form molecular iodine, I_2_, after several intermediate reactions. In 1995 Mosinger and Mosinger wrote an article describing a rapid and sensitive ^1^O_2_ assay which observed the formation of I_2_/I_3_^–^ as an indicator for ^1^O_2_ formation.^22^ In the procedure, ^1^O_2_ and I^–^ first react to form IOO^–^ and its conjugate, IOOH. This product further reacts with I^–^ to form HOOI_2_^–^, which can then dissociate into I_2_ and HO_2_^–^. Thus, both I_2_ and H_2_O_2_ (in equilibrium with HO_2_^–^) are formed under these conditions, both of which are commonly used as oxidizers for disinfection. Surprisingly, this method and its chemistry were overlooked by investigators working with fullerene materials for the production of ^1^O_2_ for many years. Only recently a group studying porphyrins and fullerenes for photodynamic therapy recognized this chemistry as a potential cause of a potentiation effect they observed with the addition of potassium iodide.^23−25^

Early reports of highly photoactive cationic fullerenes generated understandable excitement over their potential for application and concern for their fate and toxicology in the environment.^6,7,9,11^ The use of I^–^ counterions for these materials, however, significantly complicated the respective photochemical systems. These reports likely misinterpreted, and thereby misreported, experimental results by assuming that ^1^O_2_ was produced effectively by the materials (not accounting for HA effects) and that ^1^O_2_ was the oxidant responsible for observed reactions. Critical questions arise from this situation: was the contribution of ^1^O_2_ over- or underestimated in these systems? Which reactive species are present and important in such systems? Are there new opportunities or revisions that should be considered?

Only recently, investigations into the potentiation of photodynamic therapy by iodide provided some insight into the photochemistry at play. In 2018 Huang et al. showed that a delayed disinfection effect could be observed for bacterial inactivation hours after irradiation was halted, indicating a longer-lived reactive species than ^1^O_2_: they suggested hypoiodite as the culprit.^26^ Others suggest the direct reaction between ^1^O_2_ and I^–^ and subsequent formation of I_2_ and H_2_O_2_ explains potentiation observed in dye-based photodynamic therapy systems.^27^ While useful, neither of these explanations are sufficient to answer the questions posed.

It remains unclear if—or to what extent—the potentiation by I^–^ recently observed in photodynamic systems is related to HA effects, I^–^ redox chemistry, or reactions with ^1^O_2_. This knowledge is necessary to determine the degree to which prior reports on cationic fullerenes misascribed the underlying photochemical processes involved. Therefore, the present work probes the photochemical processes in cationic fullerene systems in the presence and absence of I^–^ to establish a quantitative understanding of the key reaction pathways.

## 2 Results and Discussion

### 2.1 Fullerene Aggregate Characterization

Aqueous fullerene aggregates of C_60_, C_60_-FP-SO_4_, and C_60_-FP-I were prepared by sonication and characterized using DLS and PALS after filtration. The resulting size distribution of the colloids were polydisperse with average sizes between 140 and 240 nm (see Table 1). These observations are in-line with prior studies of fullerene aggregates, which used transmission electron microscopy to reveal that these ∼150–200 nm agglomerates tend to comprise smaller units of fullerene clusters.^28,29^ Likewise, visible and UV absorption spectra of fullerene aggregate solutions (Figure S2) showed characteristic UV absorption with a tail extended into the visible range. Based on these observations, the different anions present during preparation of nC_60_-FP-I and nC_60_-FP-SO_4_ did not appear to affect the aggregate formation process or resulting physical characteristics of the particles.

**Table 1:** Hydrodynamic Diameter and ζ-Potential Values of Fullerene Aggregates with Standard Error Values of the Measurements

Sample	ζ-potential (mV)	SE (mV)	Dia. (nm)	SE (nm)
nC_60_	–28.2	1.70	141	9.85
nC_60_-FP-I	3.47	0.938	235	6.32
nC_60_-FP-SO_4_	22.0	1.79	171	3.81

### 2.2 Excited Triplet Fullerene Lifetimes

**Figure 1:**
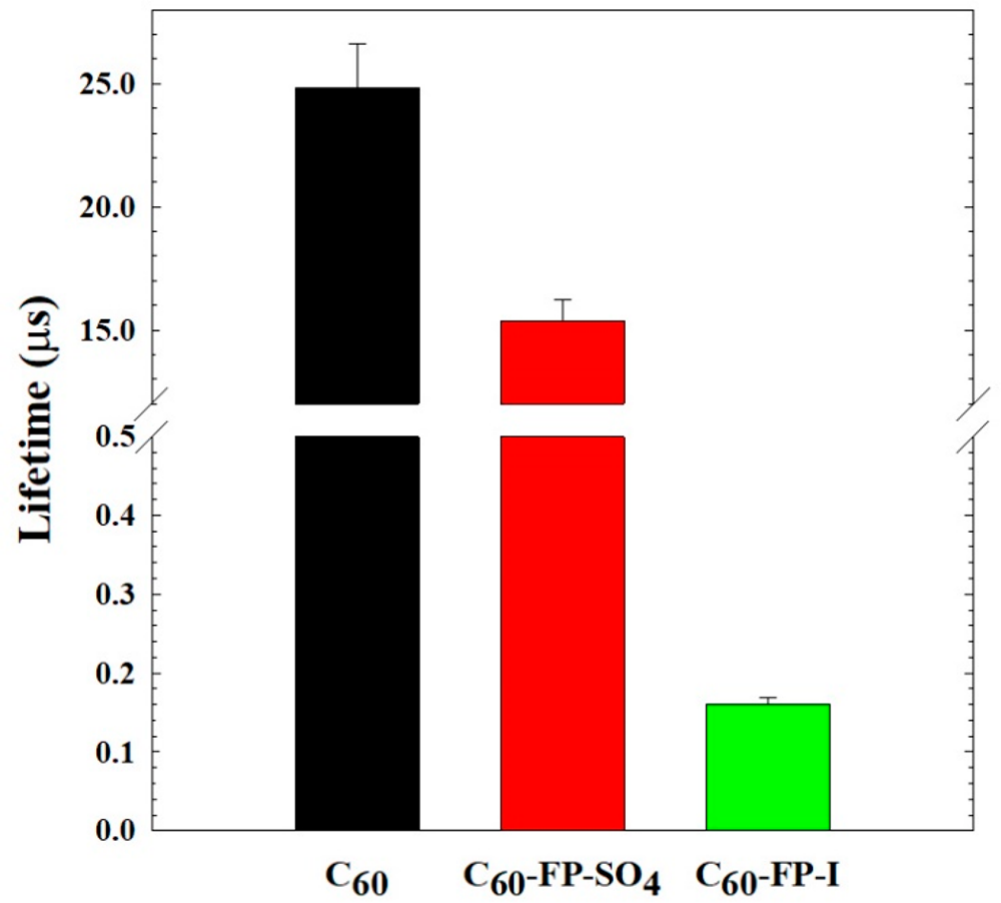
Triplet Lifetimes for fullerene in toluene (Ar sparging) and its derivatives in DMSO (5% O_2_ sparging). In an organic solvent, like DMSO, the fullerene does not aggregate and is instead simply dissolved.

Triplet excited state lifetimes of C_60_ and the C_60_-FP derivatives were measured using LFP to determine if the presence of iodide affected the triplet quenching rates. Upon photoexcitation, fullerenes rapidly undergo intersystem crossing to form a longer-lived triplet excited state, a transient species which can be observed on nanosecond time scales. Figure 1 shows the triplet lifetimes of fullerenes dispersed in organic solvents: C_60_ in toluene (Ar sparging) and C_60_-FP-I and C_60_-FP-SO_4_ in DMSO (5% O_2_ sparging). Transient profiles and corresponding fits used to calculate the triplet lifetimes are shown in Figure S3. C_60_’s excellent photoexcitation quantum yield and rapid, subsequent intersystem crossing yields a long-lived ^3^C_60_*, observed here with a lifetime of 24.84 (±1.8) μs. C_60_-FP-SO_4_, dissolved in DMSO, had a triplet lifetime of 15.38 (±0.87) μs. The presence of I^–^ in C_60_-FP-I, however, caused an approximate 100-fold reduction in lifetime, down to 0.16 μs. The drastic change here clearly indicates that I^–^ participates in ^3^C_60_* quenching. Two possible routes could result in this strong quenching of triplets; direct oxidation of I^–^ by excited fullerene or the HA effect. In either case, this observation provides strong evidence for the involvement of I^–^ in the quenching of triplet excited fullerenes.

**Figure 2:**
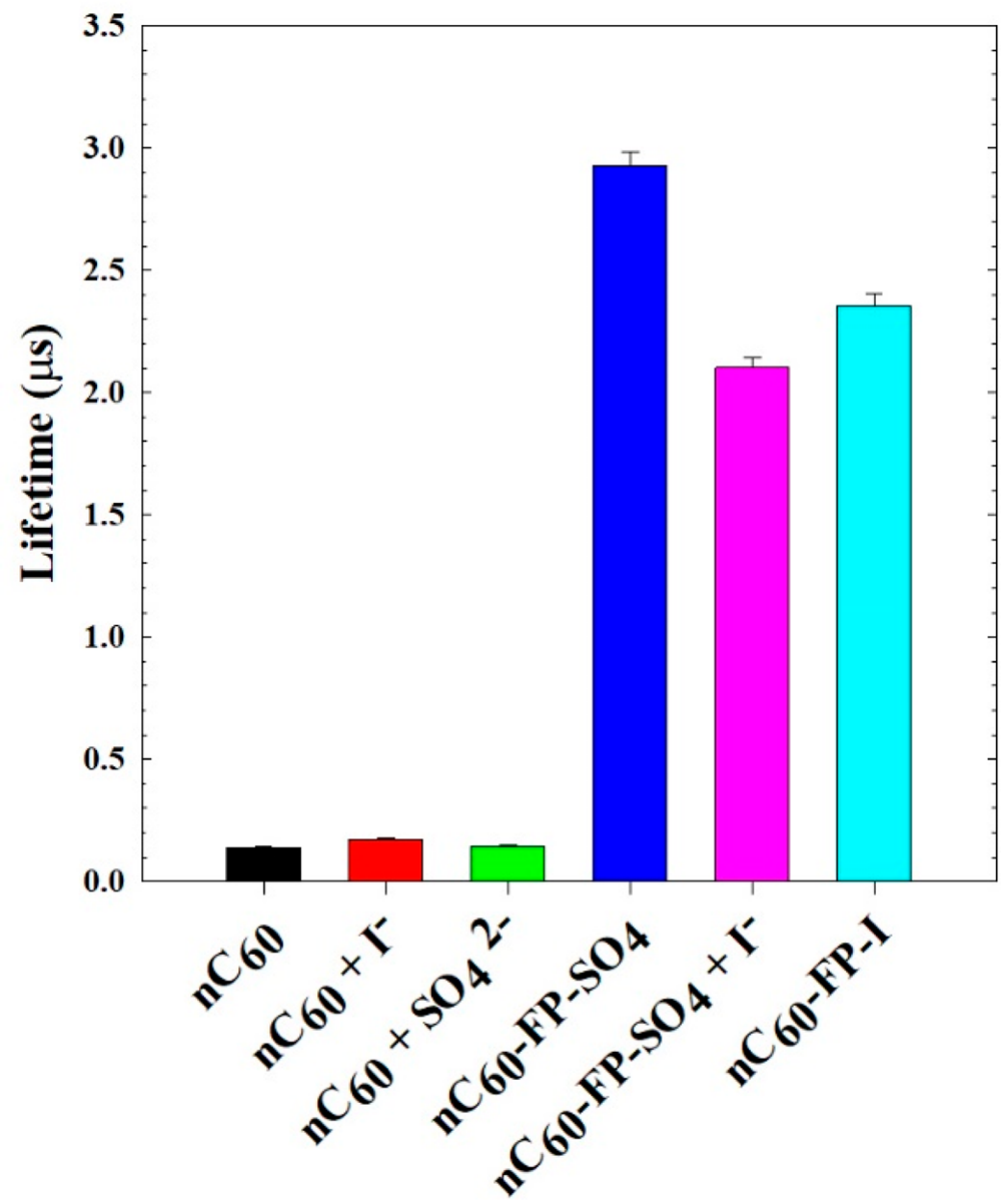
Triplet lifetimes for fullerene and fullerene derivatives in water (20% O_2_ sparging). The solutions contained 5.0 μM of nC_60_. KI was added in excess (4.76 mM) for solutions containing it.

Solutions of nC_60_ had short triplet lifetimes of 0.14 μs (Figures 2 and S3), as expected based on prior reports which have thoroughly documented the near total reduction of photoactivity of C_60_ upon aggregation in water (nC_60_).^1,3,30^ The addition of SO_4_^2–^ or I^–^ to nC_60_ solutions did not affect the triplet lifetimes, which were 0.15 and 0.17 μs, respectively. The rapid quenching of triplets in nC_60_ has been attributed to triplet–triplet annihilation of tightly aggregated fullerenes or alternately to potential back-electron transfer reactions caused by the tight solvent structure of water around C_60_–O_2_ exciplexes.^31^ Considering these factors, it was no surprise that the addition of SO_4_^2–^ or I^–^ caused no changes to the triplet lifetime of nC_60_ solutions. The presence of additional ions in solution did not observably alter the triplet quenching mechanism in nC_60_. This phenomenon is largely the reason researchers employed cationic functional groups to C_60_: to improve aqueous solubility and mitigate self-quenching. Here, the fulleropyrrolidine functionality achieved this purpose, yielding a triplet lifetime for nC_60_-FP-SO_4_ of 2.93 μs. The presence of I^–^, either by addition of KI to nC_60_-FP-SO_4_ or by the nature of nC_60_-FP-I, resulted in significantly reduced lifetimes, down to 2.35 (±0.059) and 2.10 (±0.043) μs, respectively. These lifetime reductions are considered to be caused by I^–^, considering the measurement uncertainties were small. KI was added to the nC_60_-FP-SO_4_ in excess (4.76 mM) to show the extent of the effects. The activity of I^–^ appears to exert a substantial effect on the photochemistry aqueous cationic fullerene aggregates.

### 2.3 Singlet Oxygen Production and Quenching

**Figure 3:**
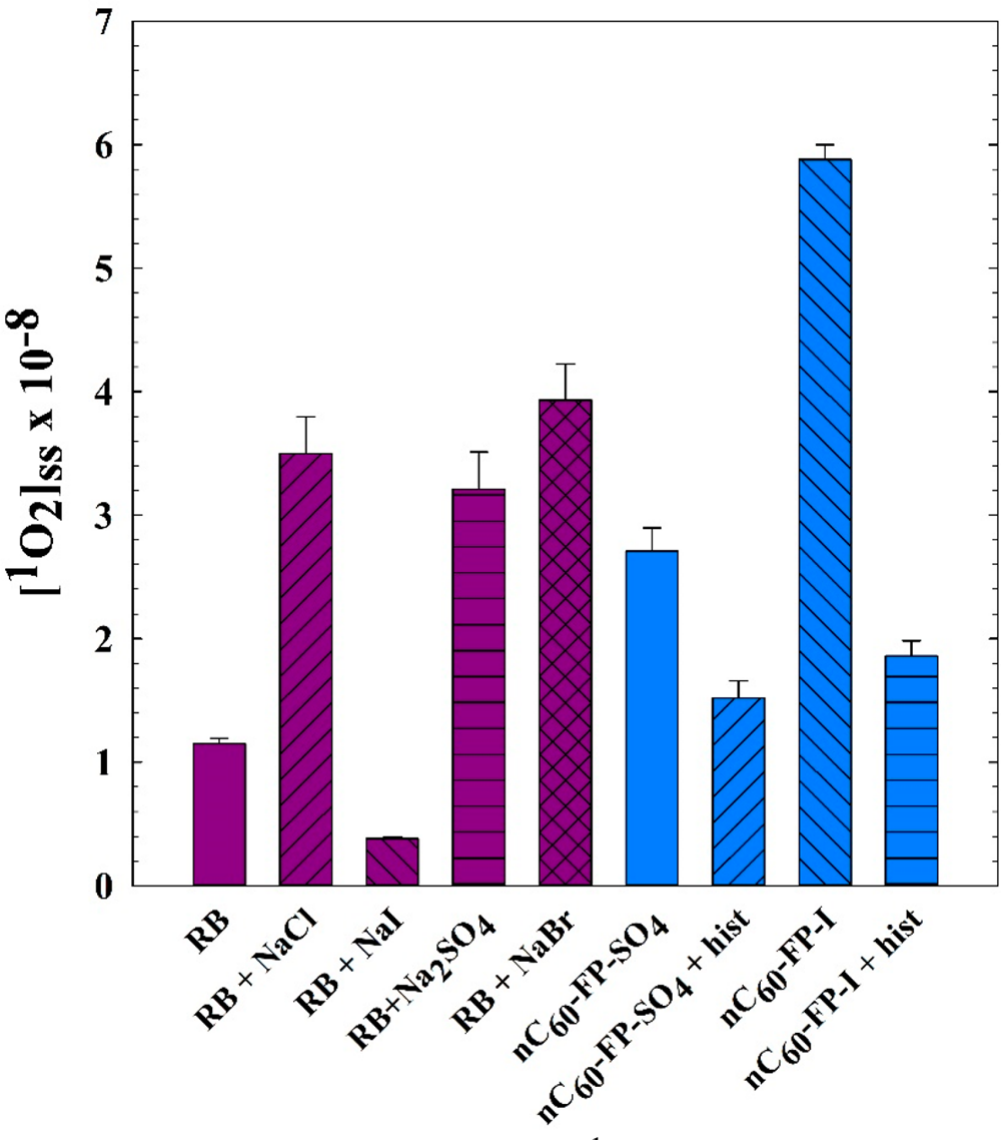
Estimated steady-state ^1^O_2_ molar concentrations for 25 μM RB or fullerene aggregates under several conditions: no additional constituents; 75 μM NaCl; 75 μM NaI; 75 μM Na_2_SO_4_; 75 μM NaBr; and 50 μM l-histidine.

Singlet molecular oxygen was presumed by many researchers to be the key reactive species relevant to fullerene photochemistry in aqueous systems, at least in the absence of electron donors. Given FFA’s well-known and specific reaction with ^1^O_2_,^32,33^ it is commonly used to estimate the steady state [^1^O_2_] achieved by fullerene photosensitization.^3,6,10,28,34^Figure 3 shows a comparison of observed [^1^O_2_]_ss_ (steady-state ^1^O_2_ molar concentration) values for fullerene and RB experiments along with l-histidine controls for each. Kinetic profiles for FFA degradation used to calculate these values are shown in Figure S4, along with FFA degradation in DMSO. Ionic species may impact the system in two ways: by altering ^1^O_2_–FFA reaction rate constants^32^ and by competitive exciplex formation between the ^3^sens* and the ion (especially anions).^18^ Rate constants for the ^1^O_2_–FFA reaction in water were computed based on temperature (25 °C) and ion content, when possible, according to Appiani et al. (2017);^32^ these values are collated in Table 2. Adding salt to RB reaction solutions improved apparent ^1^O_2_ yields in all cases except for NaI, which caused more than a 2-fold reduction in the rate of FFA loss. In contrast, the presence of I^–^ in the nC_60_-FP-I experiment either elevated [^1^O_2_]_ss_ or otherwise accelerated the destruction of FFA, significantly above that of nC_60_-FP-SO_4_. At first blush, this observation contradicts evidence of I^–^ mitigating ^3^C_60_* sensitization: the reduced triplet lifetimes, the reduced FFA photodegradation by RB with NaI, and a prior report that NaI suppressed ^1^O_2_ production.^35^ The compositions of the sensitizer molecules may play a role in the difference, because RB comprises I atoms and is not expected any further benefit from the HA effect with additional I^–^ in solution, whereas the fullerenes do not contain I atoms. Increased ^1^O_2_ yield based on the HA phenomenon does not provide a satisfactory explanation, however, because the HA effect enhances photosensitizer ^1^O_2_ yield by assisting the ISC (^1^sens* → ^3^sens*) process, which is already highly efficient for fullerenes, with ^1^O_2_ quantum yields approaching unity without heavy atoms in nonpolar solvents.^36^ Alternately, anion-sensitizer exciplexes may also form and quench the excited singlet: the HA effect is thought to accelerate quenching via nonradiative dissipation of the energy.^18^ In sum, the HA effect may play a role in the quenching of triplet excited sensitizers, but the improved FFA degradation in the fullerene case requires further elucidation.

**Table 2:** Solute-Specific FFA-^1^O_2_ Reaction Rate Constants

Salt or counterion	Concn (μM)	*k*_rxn,FFA_ (M^–1^ s^–1^)
NaCl	75	9.73 × 10^7^
NaI	75	1.04 × 10^8^^a^
Na_2_SO_4_	75	9.78 × 10^7^
NaBr	75	9.73 × 10^7^
SO_4_^2–^	37.5	9.78 × 10^7^
SO_4_^2–^	100	9.78 × 10^7^
I^–^	50	1.04 × 10^8^^a^
I^–^	75	1.04 × 10^8^^a^
I^–^	100	1.04 × 10^8^^a^

a Default used in the absence of known values.

Two alternate photochemical pathways remain as plausible explanations to the ^1^O_2_ yield observations: (1) I^–^ reacts directly with ^3^C_60_-FP*, quenching the triplet state to form I-radicals and O_2_^•–^ (and thereby H_2_O_2_ and I_2_); or (2) photosensitized ^1^O_2_ rapidly reacts with I^–^ to yield I_2_ and H_2_O_2_. When the degradation of FFA is considered, H_2_O_2_ and O_2_^•–^ are unlikely to contribute significantly,^37^ and little or nothing is known about the potential for I-radicals to react with FFA. The production of I_2_ by either route, however, could cause the observed removal of FFA, as molecular iodine is a known activator for FFA polymerization reactions.^38^ The addition of excess l-histidine, which rapidly quenches ^1^O_2_, reduced the rate of FFA removal with and without I^–^ present, suggesting that ^1^O_2_ plays an important, even if intermediate, role in the process.

### 2.4 Reactive Iodine Species

The presence of I-radicals (I• and I_2_^•–^) was probed with DMPZ, which is a known scavenger through an addition reaction that forms I-DMPZ. Despite using experimental conditions in excess (300 μM I^–^, 100 μM fullerenes, and 240 min irradiation time) of the ^1^O_2_ and LFP tests where I^–^ was shown to be impactful, no I-DMPZ was detected in experimental solutions by HPLC-UV above its LOD (0.4 μM), and no loss of DMPZ signal was observed (data not shown). The apparent lack of I-radicals in solution suggests that they are not important intermediates or end points in the photochemical system.

**Figure 4:**
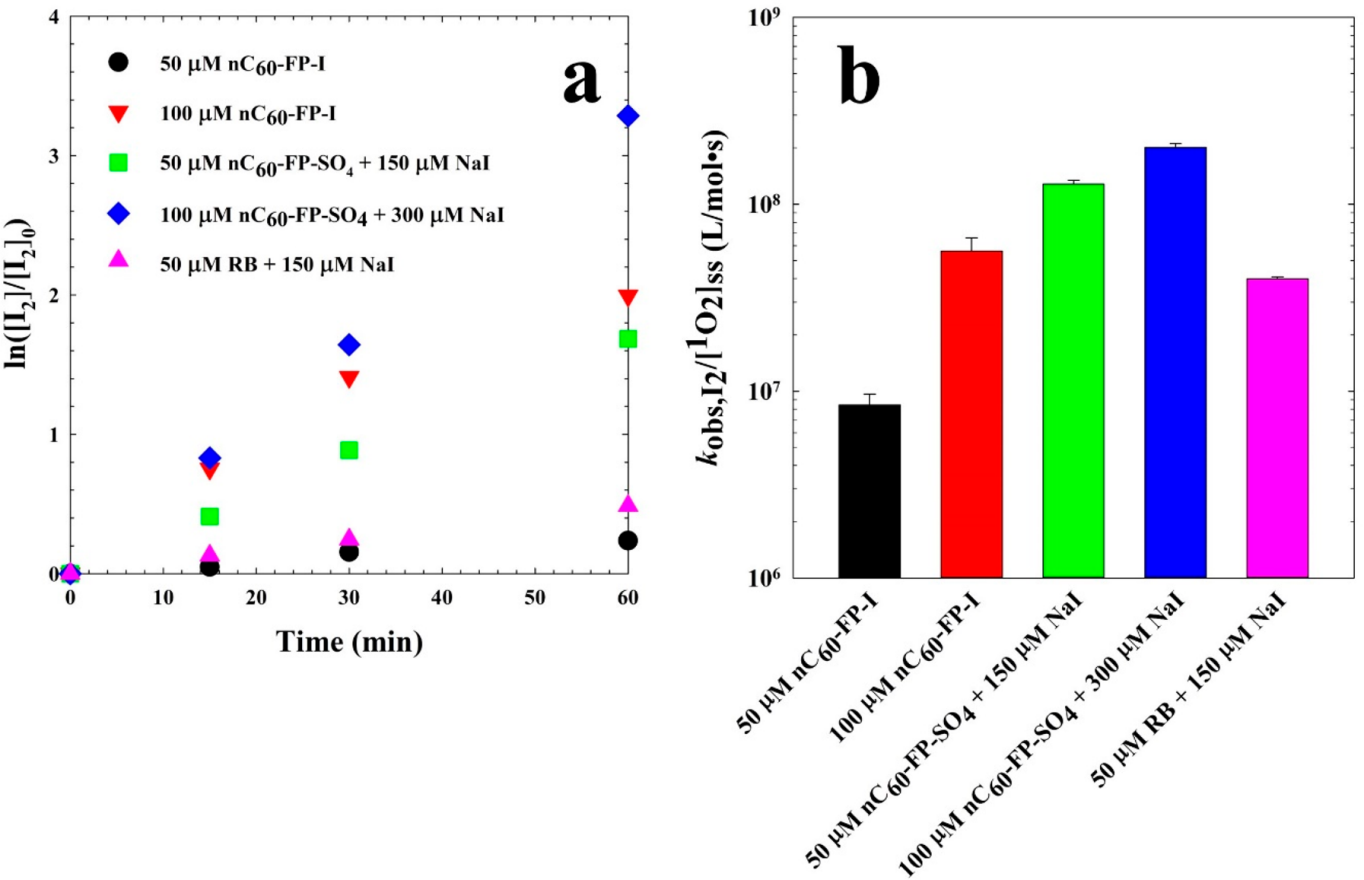
(a) Photochemical production of I_2_ over by sensitizers with NaI and 10 mM starch under UV_395_ light; and (b) observed I_2_ production yields (I_2_ production rate constants normalized by estimated steady-state ^1^O_2_ concentrations) for RB, nC_60_-FP-I, and nC_60_-FP-SO_4_.

The iodide-starch method was employed to check for I_2_ formation. Molecular iodine was found to be a significant product when nC_60_-FP was irradiated in the presence of I^–^, as shown in Figure 4. The method was sensitive down to the μM range, as shown in Figure S5. I_2_ production by the fullerenes with I^–^ in solution appeared to be approximately first-order during the initial ∼30 min (Figure 4a). The I_2_ production is particularly notable given that it was effectively photolyzed upon UV_395_ irradiation (Figure S6). There were several cases where no iodine–starch complex was observed: the obvious case with no I^–^ present, in a N_2_-purged solution (Figure S6), and upon addition of TEMPO (Figure S7). TEMPO is a known scavenger of reactive species, including ^1^O_2_ and I•, among others.^28,39^ The lack of I_2_ production in solutions without O_2_ and with TEMPO suggests that the relevant photochemistry requires O_2_ and involves reactive intermediates that can be quenched by TEMPO. Direct oxidation of I^–^ by ^3^C_60_-FP* is unlikely to be an important mechanism for I_2_ formation, given that the N_2_ purged experiment yielded no I_2_.

Having precluded the I• hypothesis and the direct ^3^C_60_-FP* oxidation pathways, the remaining route would be direct reactions between I^–^ and ^1^O_2_. This notion is challenged, however, by a comparison of the I_2_ yields on a ^1^O_2_ basis shown in Figure 4b (I_2_ production rate constants normalized by apparent ^1^O_2_ concentrations). The ^1^O_2_ estimations are based on FFA degradation kinetics, which could be confounded by reactions with I_2_. This effect should be consistent across the ^1^O_2_ sensitizers, but a further control experiment was performed to clarify the situation, using deuterated water to extend ^1^O_2_ lifetimes.^40^Figure S8 shows a comparison of FFA degradation by nC_60_-FP-SO_4_ and nC_60_-FP-I in 89% D_2_O or in a DI water solution; the predominance of D_2_O impacted both photosensitizers dramatically, increasing FFA degradation rates by factors of 7.1 (±1.02) without I^–^ present and 6.5 (±0.34) with I^–^ in solution. The fact that the FFA degradation kinetics improved significantly in both cases suggests that ^1^O_2_ is indeed playing an important role in both systems, even if it acts partly as an intermediary for I_2_ formation in solutions containing I^–^. The I_2_ production rates for nC_60_-FP increased with sensitizer concentration beyond the consequent increase in ^1^O_2_. The nC_60_-FP-SO_4_ case further highlighted the lack of I_2_–^1^O_2_ correspondence, given that the I_2_ yield for nC_60_-FP-SO_4_ was an order of magnitude higher than that for nC_60_-FP-I, given the same sensitizer and I^–^ concentrations. This discrepancy may be explained by the fact that the aggregate formation process likely traps some I^–^ within the fullerene clusters during nC_60_-FP-I preparation. A comparison between nC_60_-FP-SO_4_ and RB (Figure 4b) also suggests that the I^–^–^1^O_2_ reaction pathway is not sufficiently explanatory, because the yield for nC_60_-FP-SO_4_ was much higher than that of RB or nC_60_-FP-I, all else being equal. Apparently, the molecular composition of the sensitizer may be relevant to the observed I_2_ production.

The conundrum that O_2_ was required for I_2_ production while apparent [^1^O_2_]_ss_ did not correspond directly to I_2_ yield requires an explanation more nuanced than the three hypothetical mechanisms of I^–^ photoreactivity tested here. The importance of the sensitizer type suggests that I_2_ formation, or a critical intermediate step, occurs at the sensitizer itself as a sensitizer–I^–^ exciplex. In such a system, I^–^ may be oxidized in a photoelectron transfer (PET) reaction to form a sens^•–^–I• exciplex. PET is likely to occur in this system for both sensitizers, because PET become more likely with increasing solvent polarity, with or without HAs present.^41^ For example, RB has been used as a PET catalyst for an alkylation reaction.^42^ Since no I• was observed here, this exciplex must be an intermediate with subsequent reactions involving I^–^ and either ^3^O_2_ or ^1^O_2_ to ultimately generate I_2_. Further investigation is required to elucidate the exact mechanism here, but if a PET occurs in such an exciplex, several aspects of the sensitizer nature could then explain the I_2_ yield discrepancy observed. First, it is possible that O_2_ reactions with the exciplex prevent back electron transfer (BET) reactions in the exciplex; the proclivity of the sensitizer for BET reactions would then be critical. BET rates and exciplex lifetimes depend on the chemical and surface characteristics of the sensitizer and its moieties, if applicable.^43^ Stark differences are obvious when comparing the cationic, carbon-cage fullerenes and RB, which contains several I atoms and no charge.

### 2.5 MS2 Bacteriophage Inactivation

**Figure 5:**
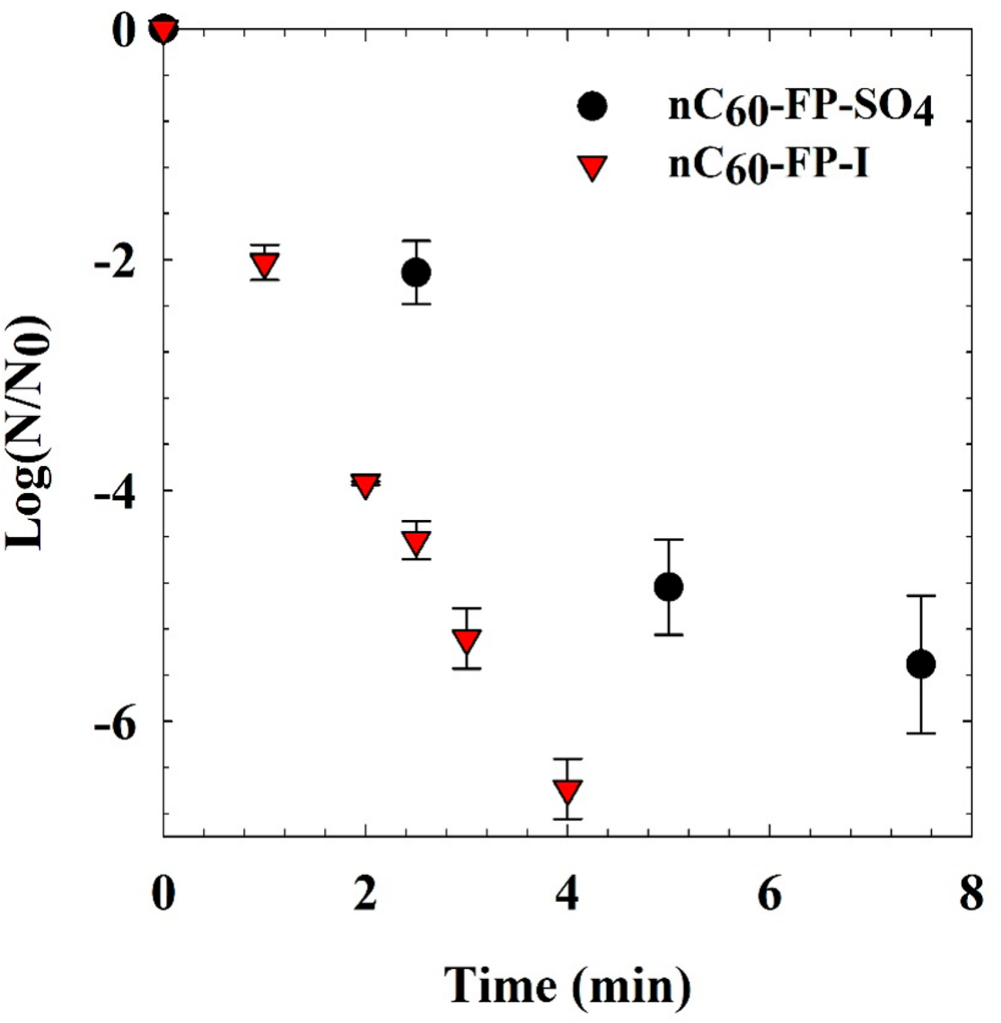
MS2 Inactivation by 250 nM of nC_60_-FP-SO_4_ or nC_60_-FP-I under visible light irradiation in DI.

The surprising observation of significant I_2_ formation in solutions of cationic fullerene derivatives calls into doubt the interpretation, if not conclusion, of many prior studies on the environmental or biological implications of these materials. The effects of ^1^O_2_ and I_2_ on MS2 bacteriophages are compared using nC_60_-FP-SO_4_ to produce ^1^O_2_ and nC_60_-FP-I, which generates I_2_ as well as ^1^O_2_ with the I^–^ present as a counterion. Figure 5 shows the results of these experiments. These materials inactivated MS2 rapidly at the low concentration of 250 nM. Prior work showed similarly rapid inactivation with the same type of fullerenes, with no MS2 inactivation under dark conditions.^10^ The low sensitizer concentration makes direct contact time comparisons between the oxidants and the rates of inactivation difficult because the methods for ^1^O_2_ and I_2_ quantification used here do not have low enough detection limits for direct observation with a 250 nM sensitizer concentration. It is clear, however, that ^1^O_2_ itself is highly effective at MS2 destruction and that the presence of I^–^ improved the MS2 inactivation kinetics further. The increase may be due to the presence of I_2_ or an increase of ^1^O_2_. Given the boost in FFA degradation shown in Figure 3 for nC_60_-FP-I over nC_60_-FP-SO_4_, what is clear is that I^–^ potentiated the important reactions. I_2_ is known to rapidly inactivate viruses,^44^ but it is unlikely that an appreciable amount of I_2_ would be generated in the bulk solution within the several minutes in this reaction. Fullerene-MS2 proximity is a likely explanation for the rapid inactivation kinetics, since MS2 and the cationic aggregates have opposite surface charges.^45^ The MS2 phage is rather susceptible to oxidation compared to bacteria eukaryotic cells, given their protective barriers.^46^ I_2_, however, is a longer-lived oxidant which is cytotoxic. The observed potentiation that others have shown for bacteria and fungi make sense in the context of photogenerated I_2_,^23,24,26^ because I_2_ can penetrate cells and cause damage from within.

## 3 Conclusions

The frequent use of I^–^ as a counterion in the preparation of cationic fullerenes used in numerous studies has inadvertently introduced a photochemically reactive species that now clouds interpretations of prior work examining functionalized fullerenes for environmental and medical applications. The potentiating effect of I^–^ in systems employing ^1^O_2_ photosensitizers was not simply a HA effect, which may indeed improve the yields of certain sensitizers. Instead, I_2_/I_3_^–^ formed during experiments here, which likely contributed to increased FFA and MS2 degradation rates. Reflecting on the literature surrounding cationic fullerenes (and possibly other photosensitizers),^6−11^ interpretations of results should attend to the counterion used in the functionalization process; those cases which used I^–^ have likely overestimated the sensitization potential of the functionalized fullerenes. Likewise, estimations of ^1^O_2_ concentrations in these systems may be faulty, given that I_2_ may react with ^1^O_2_ probe compounds. The results here suggest that studies on virus inactivation driven by I^–^-containing systems slightly overestimate the kinetics, since ^1^O_2_ alone was not as effective. For disinfection experiments on higher level organisms, the presence of I_2_ may be much more significant and is the most likely explanation of the potentiation effect observed by researchers investigating the deactivation of bacteria and fungi in medical applications.^24,25,27,47^ These observations merit consideration in the wider contexts of photochemical reactions in high-salinity systems such as engineered wastewater or marine systems, where photosensitive organics produce ^1^O_2_ in measurable quantities under UV or sunlight irradiation. Estimates of ^1^O_2_ concentrations and its relevant reactions may be misleading if the potential formation of I_2_ was not also considered. More broadly, the study of iodine geochemical cycles may benefit from scrutinizing a potential photogeochemical pathway of I_2_ formation. Global estimates of I^–^ in seawater are on the order of tens of nanomolar, which provide important sea surface reactions between I^–^ and O_3_; this interface is an important sink of O_3_ in the lower atmosphere and an exporter of reactive I_2_ into the atmosphere.^48^ Photosensitized I_2_ may play an untold role in the cycling of iodine at the sea–atmosphere interface.

## 4 Methods and Experimental Details

### 4.1 Fullerenes and Chemicals

**Figure 6:**
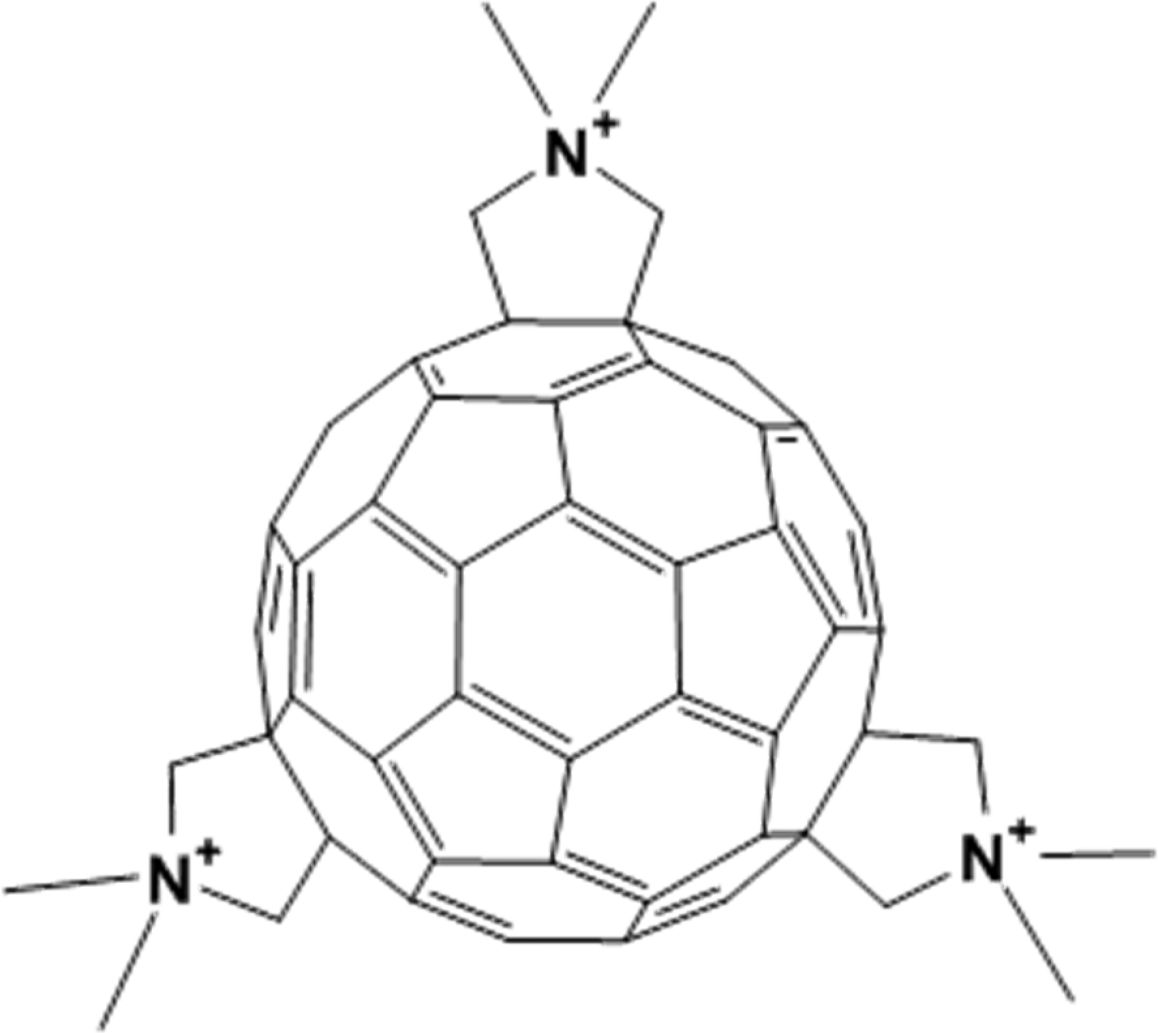
Two-dimensional illustration of the fulleropyrrolidinium ions studied here. Counterions were either I^–^ or SO_4_^2–^.

Chemicals used were reagent or biotechnic grade, according to their designated use. Dimethyl sulfoxide (DMSO), toluene, HPLC solvents, and Starch Indicator (2% w/v) were obtained from VWR International LLC (Radnor, PA). Rose Bengal (RB) and fullerene C_60_ powder (99.5% purity) were purchased from Alfa Aesar (Haverhill, MA). Deuterium oxide (99.8% D) was obtained from TCI America, Inc. (Portland, OR). Fullerene derivatives were obtained from Solaris Chem Inc. (Vaudreuil-Dorion, Quebec, Canada); these were tris-functionalized cationic fulleropyrrolidinium ions, illustrated in Figure 6, with either I^–^ or SO_4_^2–^ as counterions, labeled C_60_-FP-I and C_60_-FP-SO_4_, respectively. Ammonium molybdate (4-hydrate, reagent grade) was obtained from Ward’s Science (ON, Canada). 4-Oxo-2,2,6,6- tetramethylpiperidinooxy (TEMPO, 95%) was obtained from Across Organics (New Jersey, USA).

### 4.2 Aggregate Preparation and Characterization

Aqueous aggregates of the fullerene materials were prepared by sonication according to prior reports. Briefly, 100 mg of C_60_ were added to 25 mL of toluene and sonicated in a bath sonicator (Fisher Scientific FS30) for 24 h in a sealed jar. Next, 75 mL of nanopure water were added to the mixture and then sonicated for 24 h in a sealed jar and then further 24 h open to the atmosphere to evaporate the residual toluene. The fullerene derivatives (C_60_-FP-I and C_60_-FP-SO_4_) were directly added to nanopure water and sonicated for 24 h. The mixtures were then filtered with a 0.45 μm nitrocellulose filter. The filtered solutions were concentrated using a rotary evaporator and stored in the dark. The concentrations for the aggregates were measured using a total organic carbon (TOC) Shimadzu VCSH/CSN analyzer. The aggregated state is denoted here by adding an ‘n’ as a prefix to the label (nC_60_, nC_60_-FP-I, and nC_60_-FP-SO_4_). The fullerene aggregates were characterized for size and zeta potential using dynamic light scattering (DLS) and phase analysis light scattering (PALS), with a Zetasizer Nano ZS90 (Malvern Instruments) using a refractive index value of 2.2.^49^

### 4.3 Photochemical Experiments

Photochemical experiments were conducted in an enclosed UV cabinet with a magnetically stirred photoreactor at room temperature. An LG Innotek 6868 UV_395_ LED lamp was used as the UVA light source. The distance between the light source and reaction vessel was 20 cm. The irradiance at the location of the vessel was measured to be 9.5 mW/cm^2^ with a UVX radiometer (Analytik Jena, GmbH). Reaction solutions were kept at ambient temperature, between 24 and 25 °C, by the photoreactor cabinet. Photochemical disinfection experiments required lower intensity irradiation to adequately quantify kinetics;^10^ so, a polychromatic, visible light source was used for these tests: a 3000K lamp (Brizled Inc., DDL6) provided illumination with an incident intensity of 6.49 × 10^–7^ einsteins/min.

Furfuryl alcohol (FFA) was used at an initial concentration of 0.2 mM as a singlet oxygen probe for the sensitizer experiments. FFA been shown to be stable under UV_365_ irradiation of similar intensity as the 395 nm source used here over the experimental time frames used here.^28^ 3,5-Dimethyl-1*H*-pyrazole (DMPZ) is used as a selective probe for iodine radicals. DMPZ is a synthetic chemical which is susceptible to halogenation under UV light and combines to form the respective halo-DMPZ as 4-halo-3,5-dimethyl-1*H*-pyrazoles (XDMPZs) in products.^50^ FFA, DMPZ, and I-DMPZ were analyzed using an Agilent 1260 Infinity II High Pressure Liquid Chromatography–UV (HPLC-UV) equipped with an Agilent Eclipse Plus C18 column (3.0 mm × 150 mm, 3.5 μm). FFA was measured at 216 nm using a 10:90 mixture of 10 mM phosphoric and acetonitrile. For DMPZ and I-DMPZ, the aqueous phase was 10 mM phosphoric acid (pH 2.0) and the organic phase was acetonitrile/water (99/1, v/v) with a flow rate of 0.5 min/min. For gradient elution, the organic phase was held at 10% for 2 min, adjusted to 50% at 3 min and held until 9 min, then returned to 10% at 9.2 min. UV absorbance at 236 nm was used to quantify DMPZ and I-DMPZ, which had retention times of 2.3 and 7.9 min. The limit of detection (LOD) of I-DMPZ was 0.4 μM.

Starch indicator was used for the detection of I_3_^–^ formation in fullerene and RB experiments. Fullerene and RB solutions with starch indicator were irradiated with UV_395_ light. These solutions were diluted at a 1:4 ratio with DI water in a cuvette, and their UV/vis spectra were collected using a UV/vis spectrophotometer. The starch method forms complexes between I_3_^–^ and starch, which was quantified according to its absorption peak at 560 nm. The starch complex signal was calibrated via standard curve to provide iodine quantification in terms of total I_2_ (Figure S1).

### 4.4 MS2 Bacteriophage Culturing and Plaque Assays

Virus disinfection experiments were performed using a plaque assay method for MS2, as described previously.^6,10^ In brief, the assay coincubates the bacteriophages with *Escherichia coli* (C-3000, ATCC 15597) in a soft agar layer poured onto a hard agar layer after inoculation. The plates are then allowed to solidify prior to incubation at 37 °C overnight. Disinfection experiments were performed in triplicate under visible LEDs with 250 nM of either nC_60_-FP-I or nC_60_-FP-SO_4_.

### 4.5 Laser Flash Photolysis

Pump–probe transient absorption spectroscopy was conducted to monitor fullerene and fullerene derivative triplet excited state lifetimes using a previously described laser system.^51^ We conducted measurements with fullerene solutions in organic solvents (toluene or DMSO) at concentrations of 50 μM and in water as aggregates (preparation described above) with gas sparging with Argon, 5% O_2_, or 20% O_2_. We excited fullerene solutions with 365 nm pump excitation with a power of 3 mW and observed the broad fullerene transients formed between 570 and 750 nm. We determined transient decay lifetimes from monoexponential decay fits of the transients calculated with Surface Explorer (Ultrafast Systems, Sarasota, FL, USA) at 750 nm for C_60_ in toluene, 600 nm for the C_60_ derivatives in DMSO, 570 nm for aqueous nC_60_, and 650 nm for aqueous C_60_ derivatives, each according to maximum transient absorption. To measure the effects of added anions, we spiked I^–^ and SO_4_^2–^ into fullerene solutions from concentrated stocks and observed the changes in the transient lifetimes.

## Supporting Information Available

Figures of UV–vis spectra of iodine-starch complex and fullerene solutions, transient absorption decay profiles for LFP experiments, FFA degradation kinetic profiles, iodine-starch calibration curve, and iodine formation control experiments. (PDF)
