# Supplementary material for: The Overlooked Photochemistry of Iodine in Aqueous Suspensions of Fullerene Derivatives
Source: ACS Nano. 2022 May 9;16(5):8309–17. doi: 10.1021/acsnano.2c02281 (PMC9134498; doi:10.1021/acsnano.2c02281)
Supplement: Supplementary file 1 — nn2c02281_si_001.pdf [file nn2c02281_si_001.pdf]

Supporting Information

Title: The overlooked photochemistry of iodine in aqueous suspensions of fullerene derivatives

Authors: Madhusudan Kamat[1], Kyle Moor[2,3], Gabrielle Langlois[1], Moshan Chen[4], Kimberly M. Parker[4], Kristopher McNeill[2], Samuel D. Snow[1*]

[1]Department of Civil and Environmental Engineering, Louisiana State University, 3255 Patrick Taylor Hall, Baton Rouge, Louisiana 70803, United States.

[2]Department of Civil and Environmental Engineering, Utah State University, 4110 Old Main Hill, Logan UT 84322-4110, United States.

[3]Department of Environmental Systems Science, ETH Zurich, Universitaetstrasse 16, 8092 Zurich, Switzerland.

[4]Department of Energy, Environmental, & Chemical Engineering, Washington University in St. Louis, 1 Brookings Drive, St. Louis, MO 63130-4899, United States.

*Corresponding author: ssnow@lsu.edu;

Contents
Number of pages: 7
Figures: 8

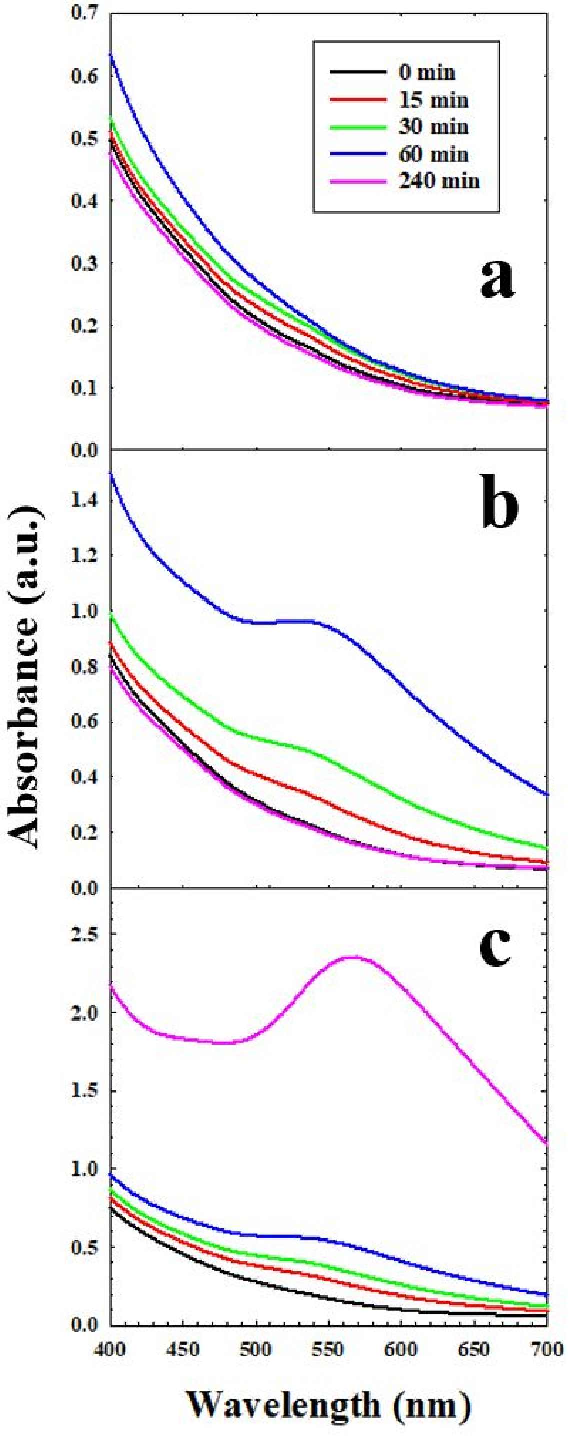

Figure S1. UV-Vis spectra of **a**) I_2 formation from irradiation of 50 μM nC_{60}-FP-I by UV_{395} in the presence of 10 mM starch; **b**) 100 μM nC_{60}-FP-I over 4 h; **c**) for 100 μM nC_{60}-FP-SO_4 in the presence of 75 μM NaI and 10 mM starch under UV_{395} irradiation over 4 h.

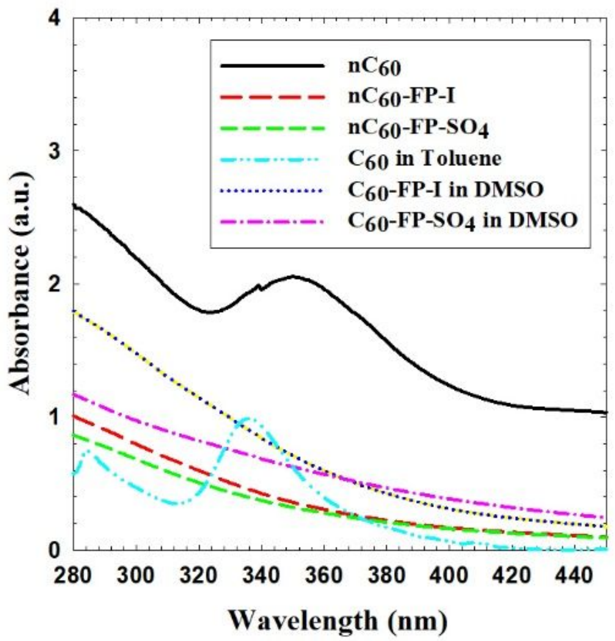

Figure S2. UV-Vis spectrum for 25 μM fullerenes dispersed in organic solvents or as aggregates in water.

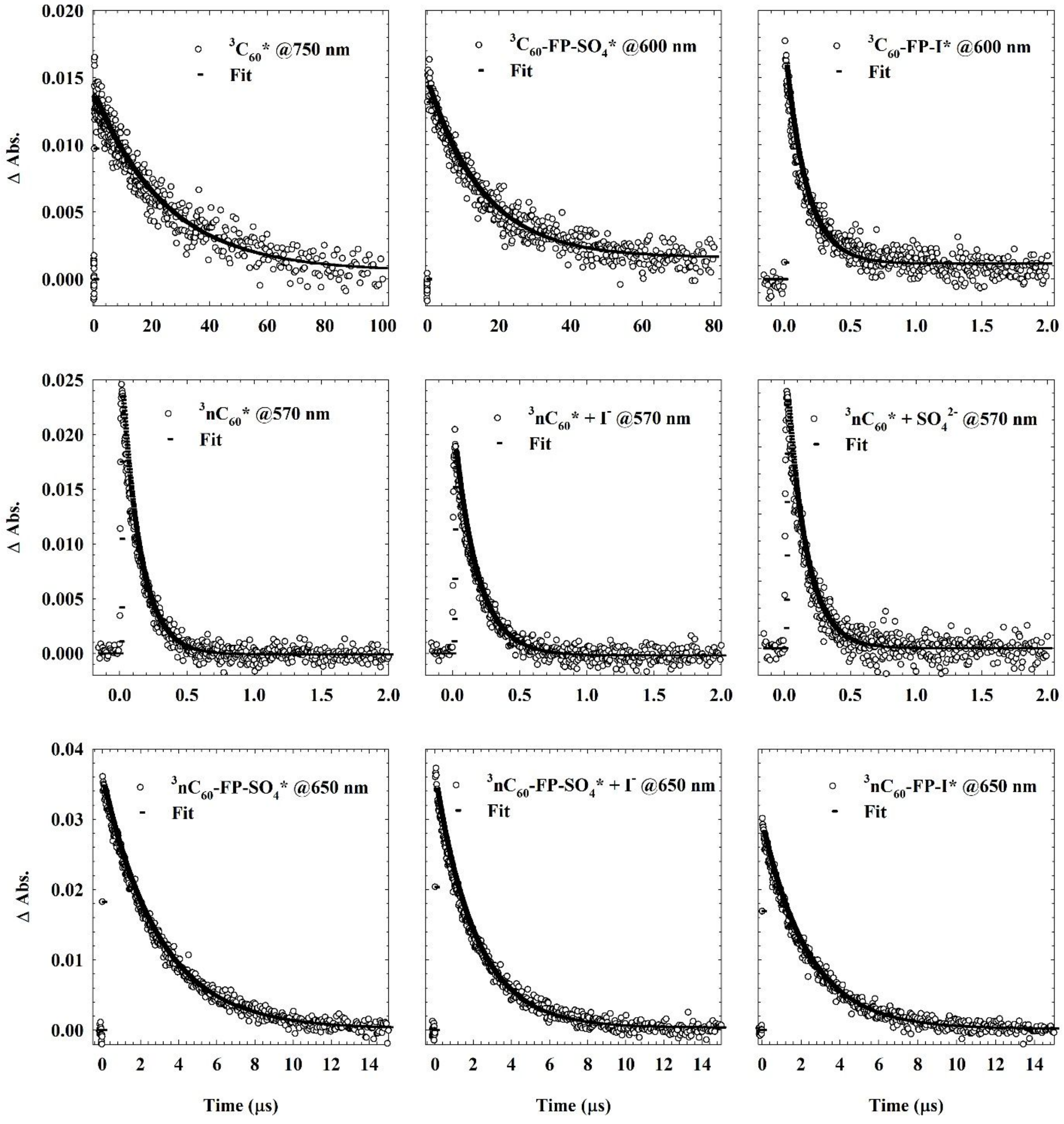

Figure S3. Transient absorption decay profiles and corresponding curve fits for C_{60} in toluene, C_{60}-FP- derivatives in DMSO, and aqueous aggregates of each with added cosolutes as shown. KI was added in excess to provide the added I-, at 4.76 mM. Absorbances were measured at optimal transient absorption wavelengths: 750 nm for C_{60} in toluene, 600 nm for the C_{60} derivatives in DMSO, 570 nm for aqueous nC_{60}, and 650 for aqueous C_{60} derivatives

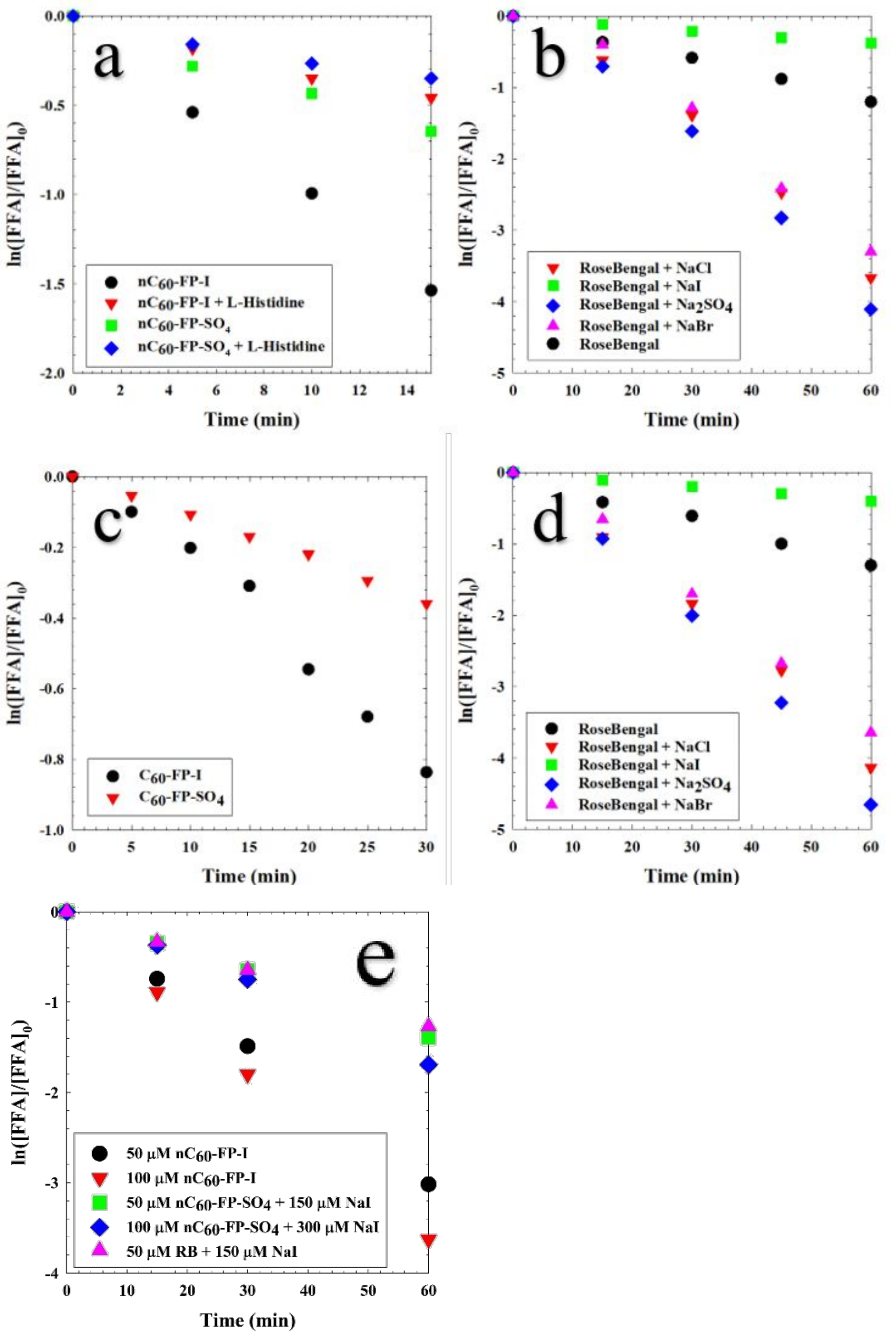

Figure S4. Photodegradation of 0.2 mM FFA under several conditions: a) in the presence of 5 μM of nC_{60}-FP-I or nC_{60}-FP-SO_4 sensitized by UV_{395} in water; b) under UV_{395} in the presence of 50 μM RB and 15 mM of various anions; c) in the presence of 5.0 μM C_{60}-FP-I or C_{60}-FP-SO_4 in DMSO; d) in the presence of 50 μM Rose Bengal and 15 mM of halide or sulfate salts under UV_{395} in DMSO; e) under UV_{395} in the presence of nC_{60}-FP-I or nC_{60}-FP-SO_4 and halide salts.

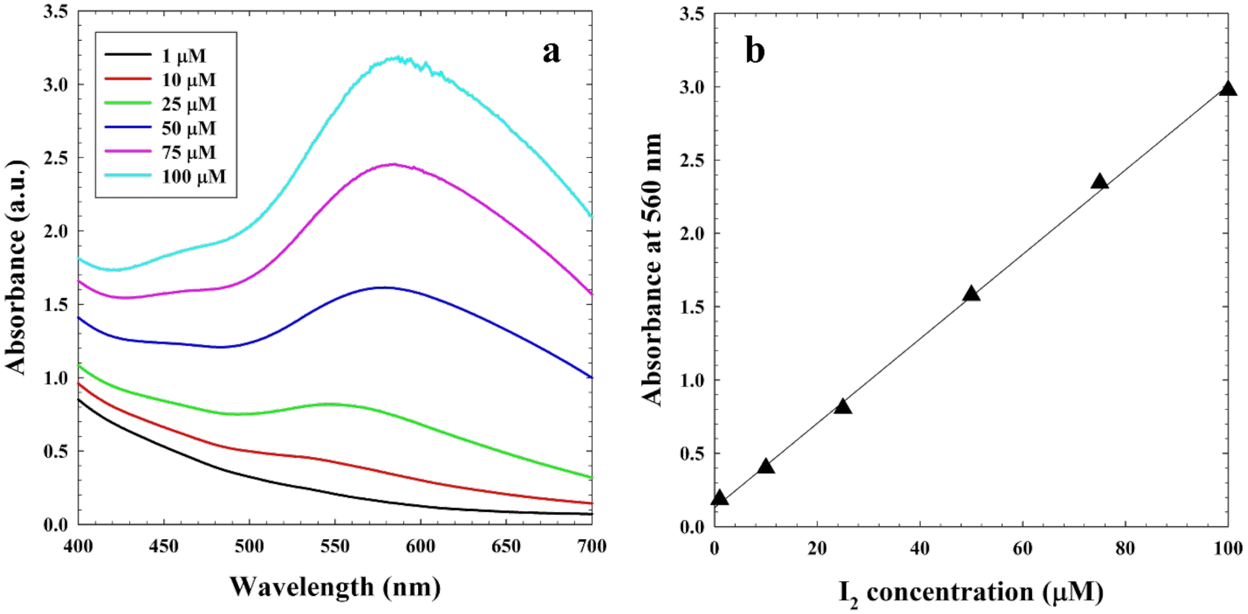

Figure S5. (a) UV-Vis spectrum for 50 μM C_{60}-FP-I and 10 mM starch with variable concentration of I_2, and (b) corresponding dose-response curve for the absorbance at 560 nm of the I_2-starch complex observed in the presence of 50 μM C_{60}-FP-I.

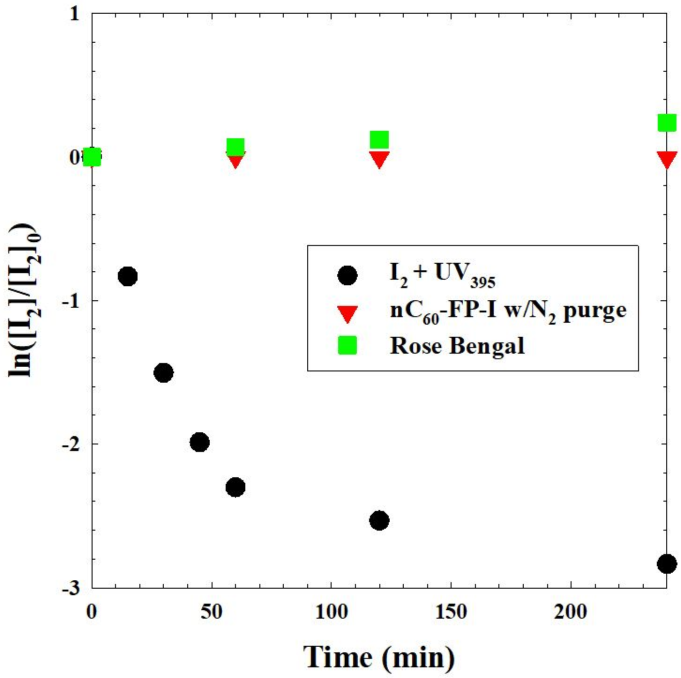

Figure S6. Photodecomposition of 300 μM I_2 under UV_{395} irradiation over 4 hours and I_2 production by 50 μM nC_{60}-FP-I in a N_2-purged solution, 75 μM NaI and 25 μM RB with no purge, measured with 10 mM starch.

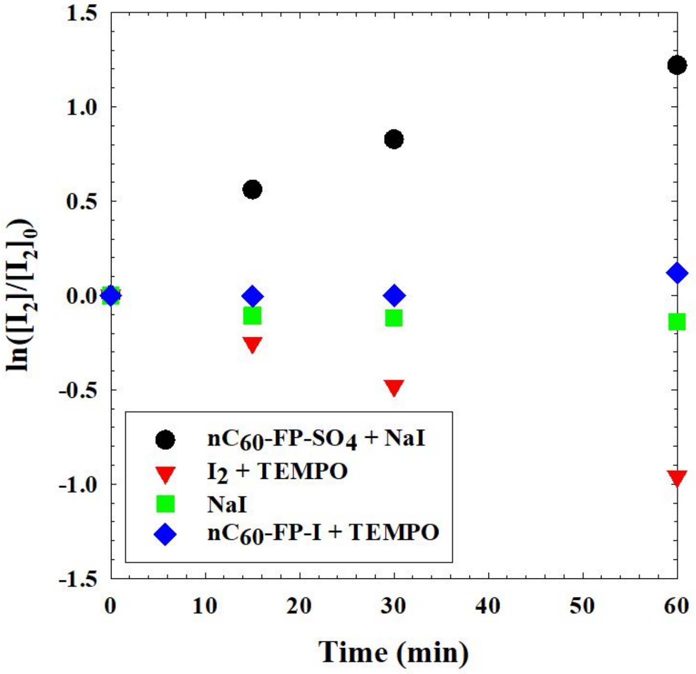

Figure S7. Formation or destruction of I_2 under UV_{395} with 100 μM C_{60}-FP-SO_4 with 75 μM NaI; 100 μM nC_{60}-FP-I with 100 μM TEMPO; 300 μM I_2 with 100 μM TEMPO; or 75 μM NaI.

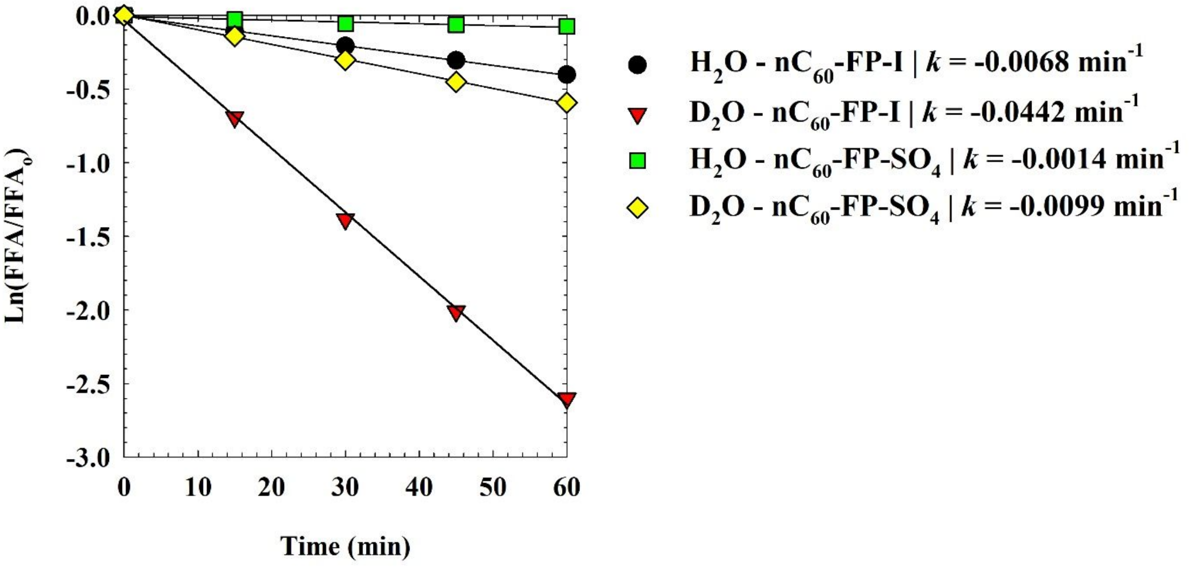

Figure S8. FFA photodegradation by 1.0 μM nC_{60}-FP-I or nC_{60}-FP-SO_4 in either DI water or 89% D_2O. Lines represent linear regressions for the experiments, with corresponding slopes provided in the figure legend.
